# Structural characterization of a new high molecular weight polysaccharide from jujube fruit

**DOI:** 10.3389/fnut.2022.1012348

**Published:** 2022-11-17

**Authors:** Xiaolong Ji, Zhiwen Wang, Xiyu Hao, Yingying Zhu, Yan Lin, Guoli Li, Xudan Guo

**Affiliations:** ^1^College of Food and Bioengineering, Zhengzhou University of Light Industry, Henan Key Laboratory of Cold Chain Food Quality and Safety Control, Henan Collaborative Innovation Center for Food Production and Safety, Zhengzhou, China; ^2^Chongqing Key Laboratory of Development and Utilization of Genuine Medicinal Materials in Three Gorges Reservoir Area, Chongqing Three Gorges Medical College, Chongqing, China; ^3^Heilongjiang Feihe Dairy Co., Ltd., Beijing, China; ^4^Basic Medical College, Hebei University of Chinese Medicine, Hebei Higher Education Institute Applied Technology Research Center on TCM Formula Preparation, Hebei TCM Formula Preparation Technology Innovation Center, Shijiazhuang, China

**Keywords:** jujube, high-molecular-weight polysaccharide, structural characterization, NMR, GC-MS

## Abstract

From *Ziziphus Jujuba* cv. *Muzao* fruit, a new polysaccharide (PZMP3-1) with high molecular weight was isolated. Constructional characterization revealed that PZMP3-1 comprized 2.56 rhamnose, 7.70 arabinoses, 3.73 galactose, and 6.73 galactose, and it has a 241 kDa average molecular weight. The principal structural components of PZMP3-1 were 1,2,4 and 1,4-linked Gal*p*A, 1,4-linked Gal*p*, 1,3 and 1,5-linked Ara*f*, and 1-linked Rha*p* based on methylation and nuclear magnetic resonance spectroscopy (NMR) analyses. X-ray diffraction (XRD), Fourier transforms infrared spectroscopy (FT-IR), atomic force microscopy (AFM), and scanning electron microscopy (SEM) structural analysis of PZMP3-1 revealed a tangled and branching pattern. Overall, these structural results suggested that PZMP3-1 could have unique bioactivities and be widely used in nutritional supplements.

## Introduction

Recent studies have investigated the biological effects of polysaccharides (PZMP3-1) that include their abilities to suppress free radicals and exert anti-bacterial, anti-cancer, anti-tumor, anti-coagulant, anti-viral, and immunological effects, etc., (1, 2). The chemical composition and structural characteristics of polysaccharides could determine their biological effects (3). Numerous studies have demonstrated that sulfation produces excellent physiological effects that support health maintenance and disease prevention (4).

The fruit of *Ziziphus Jujuba* Mill., often known as jujube, is called Jujubae Fructus and is known by the Chinese names Dazao or Hongzao (5, 6). Jujube has been demonstrated as anti-oxidant, anti-provocative, anti-microbial, anti-cancer, cardiovascular, gastrointestinal protective, anti-HIV, neuroprotective, sedative-hypnotic, anxiolytic, and other bioactivities *in vitro* and *in vivo* technological research, demonstrating the fruit’s pharmacological potential (7, 8). Bioactive metabolites are responsible for these actions, containing polysaccharides, oligosaccharides, saponins, cyclopeptide alkaloids, minerals, triterpenoid acids, vitamins, and flavonoids, which are thought to be the distinctive and functional elements of the jujube fruit (9, 10).

The origin of *Z. jujuba* cv. *Muzao* fruit is mainly from Lüliang Shanxi Province and Yulin Shaanxi Province of China (11). Polysaccharides were found in jujube’s pharmacological components and have been linked to various health benefits, such as immunomodulation, anti-cancer, anti-oxidation, hypoglycemic, hepatoprotective, and gastrointestinal protection (12, 13). Numerous investigations on low molecular weight polysaccharides have emphasized their structural characteristics and pharmacological activities (14–16). Jujube polysaccharides with molecular weights varying between 10^4^ and 10^6^ Da have been detected in various experimental conditions, and these polysaccharides demonstrated anti-oxidant activities (2, 17). From *Z. jujuba* cv. *Jinsixiaozao*, the polysaccharides (four fractions) have *Mw* values ranging from 86 to 160 kDa, according to Li et al. (18, 19). However, the higher molecular weight fraction of *Z. jujuba* cv. *Muzao* polysaccharides is not present.

In the current study, from *Z. jujuba*, isolation and purification of a unique polysaccharide with high molecular weight, was given the designation PZMP3-1. The structural conformation and physicochemical properties of PZMP3-1 were detected by model analytical instruments [gas chromatography (GC), high-performance gel permeation chromatography (HPGPC), Fourier transforms infrared spectroscopy (FT-IR), methylation analysis, X-ray diffraction (XRD), nuclear magnetic resonance spectroscopy (NMR), atomic force microscopy (AFM), and scanning electron microscopy (SEM)]. The eventual objective of this research could be to offer a new scientific understanding of the composition of polysaccharides from jujube.

## Materials and methods

### Materials

*Ziziphus Jujuba* cv. *Muzao* fruit was donated by Shaanxi Loess Plateau Experimental Orchard (China). GE Healthcare Life Sciences (Piscataway, NJ, USA) provided the Sephacryl S-300 gels and anion-exchange DEAE Sepharose Fast Flow. Sigma-Aldrich Co., Ltd (Sigma, St Louis, MO, USA) provided the standard monosaccharides. The analytical grade was used for all other compounds and reagents.

### PZMP3-1’s isolation and purification

From *Z. jujuba* cv. *Muzao*, the unprocessed polysaccharides (ZMP) were isolated by water extraction, deproteinized, decolorized, precipitated by ethanol, and freeze-dried, as shown by the method of Ji et al. (20). The dissolved ZMP in deionized water was centrifuged and then filtered the supernatant through a membrane (0.45 μm), a 2.6 × 100 cm diethylaminoethyl (DEAE)-Sepharose Fast Flow column eluted with 0.3 M NaCl was loaded with ZMP. On a 2.6 × 100 cm Sephacryl S-300 column that was equilibrated with distilled water, a separated fraction was pooled, desalted, and further purified. PZMP3-1 and PZMP3-2 were pale yellow powders and derived from the fractions of the prominent peaks collected, concentrated, dialyzed, and lyophilized to get different parts (21). For further structural characterization, the PZMP3-1 fraction was employed.

### Analysis of chemical composition

The method (phenol-sulfuric acid) detected the total amount of carbohydrates in PZMP3-1, with glucose serving as the reference (22). Bradford’s technique assessed the protein content, with bovine serum albumin as the reference (23). The Folin-Ciocalteu test assessed the total phenolic content (24). At room temperature, the UV-vis spectra of the PZMP3-1 (1.0 mg/ml) in the wavelength (200–400 nm) were captured using a spectrophotometer (25).

### Mw determination and monosaccharide analysis

The high-performance liquid chromatography (HPLC) measurement of PZMP3-1’s *Mw* was detected on an Agilent instrument (LC 1200, USA) with a 7.8 × 300 mm TSK-gel G3000PWxl column. Using a calibration curve, the *Mw* concerning dextran was calculated (26).

Utilizing a collection of monosaccharides as a standard, the Shimadzu GC (2014 C) with a high-performance capillary column DB-17 (30 ml × 0.25 mm ID; 0.25 μm film thickness, Agilent) measured the various monosaccharide by comparing retention durations and peak regions (27).

### FT-IR and NMR analysis

PZMP3-1 was combined with 100 mg of potassium bromide (KBr) powder before being crushed into granules for infrared spectral measurement between 4,000 and 400 cm^–1^. A spectrophotometer was used to measure the FT-IR spectra of polysaccharide (VERTEX 70, Bruker, Germany) (28).

To replace exchangeable protons, the freeze-dehydrated PZMP3-1 (50 mg) was dissolved in 2 ml of 99.9% D_2_O and freeze-dried three times. A Bruker-600 MHz NMR Spectrometer (Bruker, Rheinstetten, Germany) was used to record the one-dimensional NMR spectra for the ^1^H and ^13^C at 25°C. Using the standard Bruker NMR software, data were collected and examined (21).

### Methylation analysis

As described in previous studies (29), PZMP3-1 was methylated, followed by hydrolysis, reduction, and acetylation, to analyze glycosyl bonds. The measurement of partially methylated alder aldehyde using gas chromatography/mass spectrometry (GC-MS) requires the use of GCMS-QP2010 Ultra apparatus and a DB-17MS capillary column (60.0 m × 0.25 mm × 0.25 μm).

### Analysis of the molecular structure

PZMP3-1 used the XRD pattern to determine the crystal structure present. The angular range of the diffractometer was 5–50° (2θ), the step size was 0.01°, the scan speed was 15°/min, and tube pressure (40 kV) and tube flow (40 mA) were present. Using an SEM (S-4800, Japan), the morphological characteristics of PZMP3-1 were documented. We used the Cressington 208 HR Sputtering Coater with sputtering gold samples. We dissolved the polysaccharides with distilled water and dried the test samples dripping on the mica carrier surface at ambient pressure of 70°C. A 5500 atomic force microscope (Agilent) was used to create the AFM images (30).

### Data analysis

Version 17.0 of SPSS was used for the statistical analysis. The data were reported as mean ± standard deviation (SD), with Duncan’s multiple-range test (*p* < 0.05) arriving after the analysis of variance (ANOVA) for each experiment, which was carried out in triplicate.

## Results and discussion

### PZMP3-1’s extraction and purification

**FIGURE 1:**
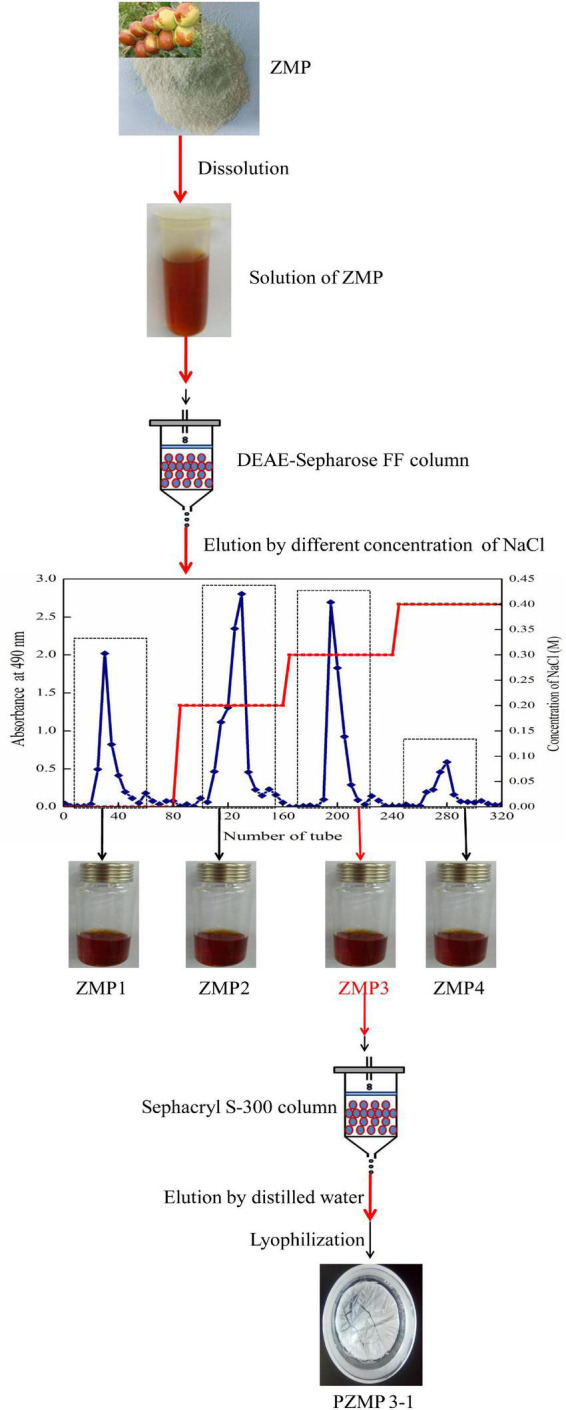
The PZMP3-1’s separation flow diagram.

In the current study, the yield of about 3.82% of the fruit of the *Z. jujuba* cv. *Muzao* was used to produce crude ZMP. On a Sepharose Fast Flow column (DEAE), the ZMP was separated using 0.3 M NaCl solution (Figure 1). This fraction was then further purified using Sephacryl S-300 columns and yielded 3.04% of PZMP3-1, the same as the neutral polysaccharide PZMP1 from *Z. jujuba* cv. (2.95%) (31).

### Characterization of preliminary PZMP3-1

**FIGURE 2:**
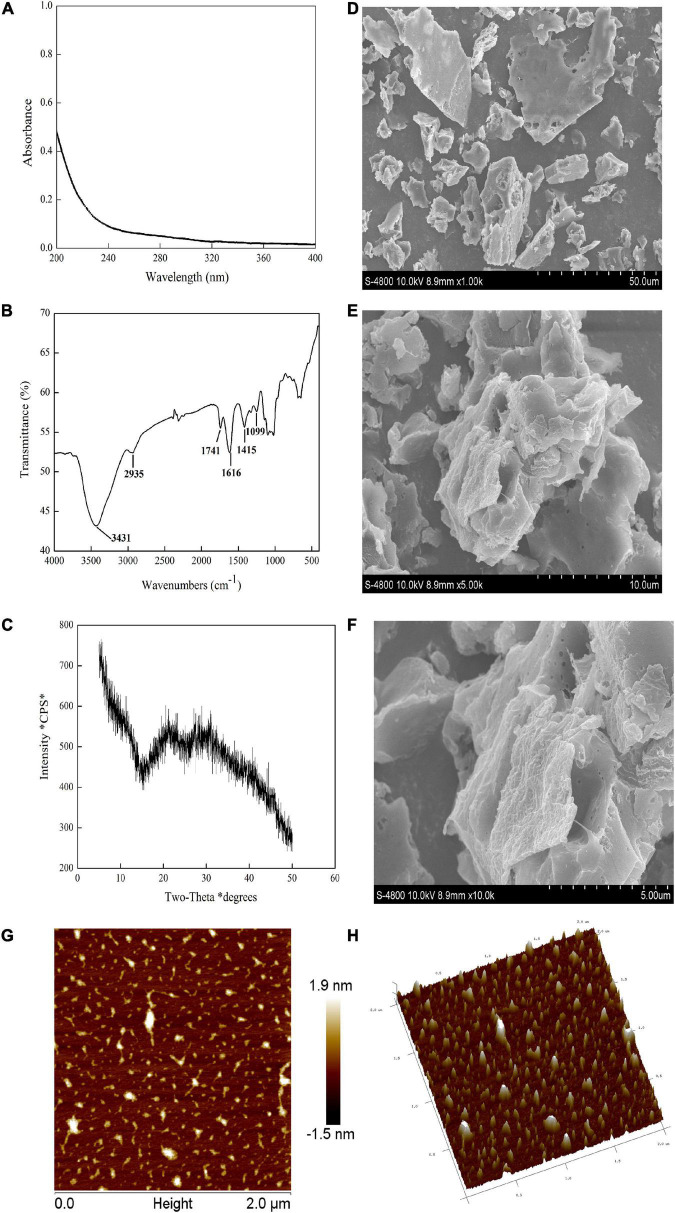
PZMP3-1’s physicochemical analysis. **(A)** The X-ray diffraction (XRD) pattern. **(B)** The spectrum of UV-vis. **(C)** The spectrum of Fourier transforms infrared spectroscopy (FT-IR). **(D,E)** Scanning electron microscopy (SEM) images [×1,000 in panel **(D)**, ×5,000 in panel **(E)**, and ×10,000 in panel **(F)**]. **(G)** Atomic force microscopy (AFM) images; **(H)** a three-dimensional image of AFM.

The number of carbohydrates in PZMP3-1, followed by the phenol-sulfuric acid method, was 95.35 ± 1.25%. The result was different with *Z. jujuba* cv. *Hamidazao* polysaccharides which Yang et al. reported that only 0.32% polyphenol and 2.11% protein were found (14). Figure 2A demonstrates that the lack of nucleic acids and proteins is shown by PZMP3-1’s UV spectrum (there is no absorption at 260–280 nm), which is in line with the research of chemical analysis as previously reported (32).

### Determination of Mw and monosaccharide compositions of PZMP3-1

Biological activity depends on the *Mw* distribution of plant polysaccharides (33). The *Mw* of PZMP3-1 was determined to be 241 kDa based on the calibration curve of standards, which corresponded to a retention period of 17.10 min and might indicate a higher molecular weight polysaccharide. Li et al. observed that from *Z. jujuba* cv. *Jinsixiaozao*, the molecular weights of four polysaccharide fractions (ZSP1b, ZSP2, ZSP3c, and ZSP4b) varied between 86 and 160 kDa (19), and Cui et al. showed that Fructus Jujubae polysaccharides consisted mainly of low molecular weight (83.8 and 123.0 kDa) fractions (34). The PZMP3-1 obtained in this study significantly exceeded the polysaccharide *Mw* shown in previous studies. The *Mw* of *Z. jujuba* polysaccharide obtained by each research group was different. This may be due to the variety, extraction, purification process, and test method (13, 35).

The PMP-GC technique was used to examine the monosaccharides content of PZMP3-1. Rhamnose, arabinose, galactose, and galacturonic acid made up most of PZMP3-1, with the molar ratios being 2.56:7.70:3.73:6.73, with arabinose and galacturonic acid making up the most significant amount when compared to other monosaccharides, according to the monosaccharide standards. Previous reports (HJP1, the ratio of mannose to galacturonic acid to rhamnose to galactose to glucose to arabinose, was 1.3:6.7:27.6:13:3.7:47.6 and HJP3 was 0.6:16.7:16:21:6.5:39.2) reported by Wang et al. could be used to confirm the polysaccharide molar ratio and monosaccharide content found in *Z. jujuba* cv. *Dongzao* (36), which had rhamnose, arabinose, galactose, glucose, and xylose ratios of 1.0:3.6:1.0:0.5:0.2 (37) and had results that were distinct from those of PZMP3-1 in this work. The varietals, production conditions, and measurement techniques might play a role in the monosaccharide composition of jujube polysaccharides (13, 14).

### FT-IR spectra of PZMP3-1

Detecting distinctive organic groups in polysaccharides using FT-IR spectroscopy is a powerful technique (38, 39). The primary functional groups of plant polysaccharides may be better understood using the FT-IR spectrum. The FT-IR spectra showed that PZMP3-1 contained the typical absorption peaks of plant polysaccharides (Figure 2B; 40). Stretching vibration (O-H) caused the characteristic peak at 3,431 cm^–1^, and stretching vibration (C-H) caused the rise at 2,935 cm^–1^ (41). They were regarded as the defining bands for polymers comprized of plant polysaccharides because of their two significant absorption peaks. Stretching vibrations (carboxylic groups) were connected to the absorption peak at 1,741 cm^–1^ (29). PZMP3-1’s absorption peak at 1,616 cm^–1^ showed that symmetrical stretching vibrations (C = O) were present (42). The bands in the 1,415 cm^–1^ likely represented the bending and deformation of C-OH and C-H vibrations, respectively (43). The intense bands at 1,099 and neighboring 1,000 cm^–1^ revealed the pyranose form of galactosyl residues (44).

### Methylation analysis of PZMP3-1

Less information about the detailed structure of jujube’s high molecular weight polysaccharides is available in the literature, especially the glycosidic bond types. Methylation analysis could determine the kind and number of glycosidic linkages in plant polysaccharide polymers (45). To determine PZMP3-1’s glycosidic bond types by GC-MS analysis, it was methylated, hydrolyzed, reduced, and converted into partially methylated alditol acetates (PMAAs) (46). The linkage patterns of PZMP3-1 were compiled, which are shown in Table 1, using a spectral database based on the PMAA standard date in the Complex Carbohydrate Research Center (CCRC) spectrum database, retention duration, and relevant literature. Six different methylated sugar derivatives were found, i.e., 1,2,4 and 1,4-linked Gal*p*A, 1,4-linked Gal*p*, 1,3 and 1,5-linked Ara*f*, and 1-linked Rha*p*. The composition of monosaccharides and PZMP3-1 was discovered using methylation analysis (45). NMR spectra provided additional evidence supporting the structure of PZMP3-1.

**TABLE 1:** PZMP3-1’s methylation analysis results.

Peak no.	Residues	Retention time (min)	Methylated sugars	Linkage patterns	Relative amount (mol%)
1		40.086	2,3,4-Me_3_-Rha*p*	Rha*p*-(1→	7.45
2	C	40.245	2,4,5-Me_3_-Ara*f*	→3)-Ara*f*-(1→	5.72
3	D	41.437	2,3-Me_2_-Ara*f*	→5)-Ara*f*-(1→	39.75
4	E	63.049	2,3,6-Me_3_-Gal*p*	→4)-Gal*p*-(1→	16.5
5	A	65.037	2,4,6-Me_3_-Gal*p*A	→4)-Gal*p*A-(1→	22.93
6	B	66.250	3,6-Me_2_-Gal*p*A	→2,4)-Gal*p*A-(1→	7.65

### PZMP3-1’s NMR analysis

**FIGURE 3:**
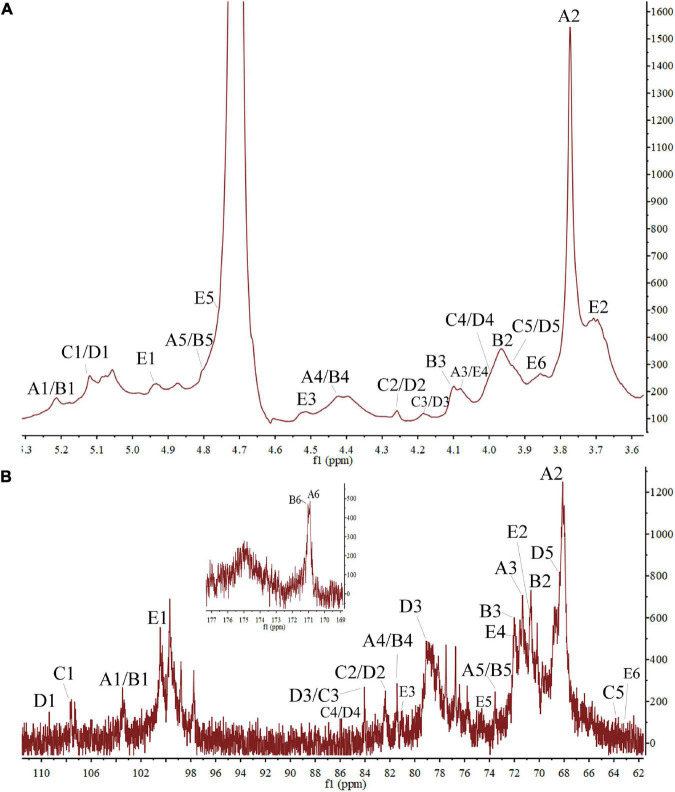
The PZMP3-1’s nuclear magnetic resonance spectroscopy (NMR) spectra in D_2_O. **(A)** ^1^H spectrum. **(B)** ^13^C spectrum.

To understand the structural characteristics of PZMP3-1, ^1^H and ^13^C NMR spectra (one-dimensional) were identified and resolved. The five residues of PZMP3-1 that GC-MS isolated were given chemical shifts (Table 2) based on 1D spectra (Figure 3) and information from the literature. Five anomeric signals were present in the ^1^H and ^13^C NMR spectra (Figures 3A,B) at 3.70–5.25 ppm and 60–110 ppm, respectively. The primary anomeric proton signals in PZMP3-1 were identified as A, B, C, D, and E in the ^1^H NMR spectra at 5.21, 5.21, 5.12, 5.12, and 4.94.

**TABLE 2:** PZMP3-1’s ^1^H and ^13^C nuclear magnetic resonance spectroscopy (NMR) data.

Residues	Linkage		1	2	3	4	5	6
C	→3)-Ara*f*-(1→	C	107.53	82.26	84.04	86.79	63.93	
		H	5.12	4.28	4.18	3.98	3.92	
D	→5)-Ara*f*-(1→	C	109.35	82.26	79.06	86.79	68.34	
		H	5.12	4.28	4.17	3.99	3.93	
E	→4)-Gal*p*-(1→	C	100.52	70.56	80.96	72.01	74.39	63.67
		H	4.94	3.71	4.50	4.07	4.77	3.85
A	→4)-Gal*p*A-(1→	C	103.39	68.08	71.26	81.37	73.49	170.91
		H	5.21	3.77	4.08	4.42	4.80	
B	→2,4)-Gal*p*A-(1→	C	103.39	70.62	71.98	81.37	73.49	171.01
		H	5.21	3.97	4.10	4.42	4.80	

In the ^1^H NMR spectrum, residues C-2, 3, 4, and 5’s protons received chemical shifts between 3.7 and 4.28. Five anomeric signals were resonated at 103.39, 103.39, 107.53, 109.35, and 100.52 after labeling the corresponding signal (allogeneic carbon) tagged in the ^13^C NMR spectra. Based on findings from the literature, Table 2 displays the results; the signals of all the tagged residues in the ^1^H and ^13^C NMR spectra are assigned. The heteropoly proton of residue A’s chemical shift caused the signal at δH 5.21, and in the heterogeneous carbon, the corresponding signal was seen at δC 103.39. The signals were created by the residue A’s C-2, 3, 4, 5, and 6 at δC 68.08/δH 3.77, δC 71.26/δH 4.08, δC 73.49/δH 4.80, and δC 170.91, respectively. According to the NMR data, this residue A’s chemical shifts were the same as those of →4)-Gal*p*A-(1→ (17). Similar to how residue B was identified as →2,4)-Gal*p*A-(1→, the signals at δC 2 70.62/δH 2 3.97, δC 3 71.98/δH 3 4.10, and δC 6 171.01 matched the residue B (anomeric carbons and protons) (47).

Ara*f* was initially given credit for the cross-peak in the anomeric area at 107–110 ppm (residues C and D). The chemical shifts of H-1, 2, 3, 4, and 5 were determined from the ^1^H spectra at 5.12, 4.28, 4.18, 3.98, and 3.92 ppm, respectively (Figure 3A), in agreement with previous reports. From the proton chemical shifts in the C spectra, the carbon chemical shifts of the residue C from C-1, 2, 3, 4, and 5 were found (Table 2). The carbons and anomeric protons of residue D were identified by the signals at δC-1 109.35/δH-1 5.12, δC-3 79.06/δH-3 4.17, and δC-5 68.34/δH-5 3.93. These outcomes corroborated FT-IR and methylation analysis results, demonstrating that residues C/D were (1→3)-linked Ara*f*/(1→5)-linked Ara*f* (29, 31). According to the NMR data, the similar alternate signals for carbon and hydrogen were 100.52 (4.94), 70.56 (3.71), 80.96 (4.50), 72.01 (4.07), 74.39 (4.77), and 63.67 (3.85), this residue’s chemical changes were the same as those of 1,4-linked Gal*p* (48, 49).

### Morphological properties of PZMP3-1

It is generally known that plant polysaccharides could be crystallized using XRD technology (50). Figure 2C depicts the X-ray diffraction pattern of PZMP3-1. PZMP3-1 had a prominent peak that appeared at around 20 and 30°, and its X-ray diffraction curves were “bun-shaped” (30, 51). The semicrystalline structure we obtained earlier could explain that PZMP3-1 might have some semicrystalline structures (an abundant polysaccharide in galacturonic acid from *Z. jujuba* cv. *Muzao*) (21, 52).

The findings of the SEM analysis of PZMP3-1’s surface morphology are shown in Figures 2D–F. PZMP3-1 was formed in an aggregation condition with an unsteady surface. The formation of intermolecular and intramolecular hydrogen bonds between polysaccharides requires the polysaccharide to have a higher molecular weight (30, 53). For this reason, PZMP3-1 revealed a tangled structure that is folded with each other.

Observing the three-dimensional structure of biologically active macromolecules requires using AFM and nanoscale microstructures, especially the form of plant polysaccharides (54, 55). PZMP3-1’s 3-dimensional and planar AFM pictures are displayed in Figures 2G,H, respectively. PZMP3-1 had a linear or branched structure, and the chain of PZMP3-1 featured a helical shape. PZMP3-1 aggregation might be explained by intermolecular and intramolecular hydrogen bonding on its surface, which acted as a catalyst to produce the potent intramolecular and intermolecular connections and the interactions with the water molecule (56, 57). Considering these morphological traits, we could conclude that the entangled and branched structure existed in PZMP3-1 molecules, which could significantly affect the bioactivity, structure, and distribution.

## Conclusion

In the current work, *Z. jujuba* cv. *Muzao* fruit was used to create a new polysaccharide PZMP3-1 with high molecular weight, and its characteristics were elucidated through physicochemical and experimental investigations using modern analytical instruments. Rhamnose, galactose, arabinose, and galacturonic acid were the main components of PZMP3-1, which was 241 kDa in weight and had a molar ratio of 2.56:3.73:7.70:6.73. PZMP3-1’s primary linkage types included →2,4)-Gal*p*A-(1→, →4)-Gal*p*A-(1→, →4)-Gal*p*-(1→, →5)-Ara*f*-(1→, →3)-Ara*f*-(1→ and Rha*p*-(1→ based on the findings of the methylation and NMR analyses. According to studies on chain conformation, PZMP3-1 was entangled with itself. The biological functions of PZMP3-1 and the links between its structure and activity are the subject of in-depth research.

## Data availability statement

The original contributions presented in this study are included in the article/supplementary material, further inquiries can be directed to the corresponding authors.

## Author contributions

XJ was involved in the study’s idea, design, and funding. ZW and XH set the database into order. YZ and YL wrote the initial draft of the document. XG participated in writing – review and editing. GL provided funding for this manuscript. All authors approved and reviewed the article’s submission.
